# A Flexible, Fireproof, Composite Polymer Electrolyte Reinforced by Electrospun Polyimide for Room-Temperature Solid-State Batteries

**DOI:** 10.3390/polym13213622

**Published:** 2021-10-20

**Authors:** Boheng Yuan, Bin Zhao, Zhi Cong, Zhi Cheng, Qi Wang, Yafei Lu, Xiaogang Han

**Affiliations:** 1 State Key Laboratory of Electrical Insulation and Power Equipment, School of Electrical Engineering, Xi’an Jiaotong University, Xi’an 710049, China; yuan980808@stu.xjtu.edu.cn (B.Y.); zhaobin87@xjtu.edu.cn (B.Z.); jycz1997@stu.xjtu.edu.cn (Z.C.); chengzhi@stu.xjtu.edu.cn (Z.C.); wq4116005096@stu.xjtu.edu.cn (Q.W.); luyafei0126@stu.xjtu.edu.cn (Y.L.); 2 Key Laboratory of Smart Grid of Shanxi Province, School of Electrical Engineering, Xi’an Jiaotong University, Xi’an 710049, China

**Keywords:** electrospinning, polyimide, solid state batteries, composite polymer electrolyte, photo polymerization, fireproof

## Abstract

Solid-state batteries (SSBs) have attracted considerable attention for high-energy-density and high-safety energy storage devices. Many efforts have focused on the thin solid-state-electrolyte (SSE) films with high room-temperature ionic conductivity, flexibility, and mechanical strength. Here, we report a composite polymer electrolyte (CPE) reinforced by electrospun PI nanofiber film, combining with succinonitrile-based solid composite electrolyte. In situ photo-polymerization method is used for the preparation of the CPE. This CPE, with a thickness around 32.5 μm, shows a high ionic conductivity of 2.64 × 10^−4^ S cm^−^^1^ at room temperature. It is also fireproof and mechanically strong, showing great promise for an SSB device with high energy density and high safety.

## 1. Introduction

Decarbonization is the global trend, which has led to considerable attention on the research of battery-energy storage systems enabling high-efficiency utilization of renewable solar and wind energy. Li-ion batteries have become dominant energy storage device in portable electronic devices and electric vehicles [1,2,3]. However, energy density and safety of the current state-of-the-art Li-ion batteries still do not meet the requirements. For this concern, Li-metal batteries with metallic Li anode have been intensively studied [4,5,6,7]. The main problems of liquid Li-metal batteries include side reaction between electrolyte and lithium metal and the growth of lithium dendrites, which can be addressed by solid-state Li-metal batteries that are considered as next generation batteries-technology [8,9,10,11].

In solid-state batteries (SSBs), solid-state electrolytes (SSEs) lie at the heart. SSEs can be summarized in three categories: inorganic solid electrolytes (ISEs) [12,13,14], solid polymer electrolytes (SPEs) [15,16,17,18,19], and composite polymer electrolytes (CPEs) [20,21,22]. For an ideal SSE, it should have high ionic conductivity, strong mechanical strength, good flexibility, wide electrochemical stability, nonflammability, etc. ISEs own the highest ionic conductivity among all types of SSEs, but they are hard to fabricate thin-film electrolyte with high flexibility and mechanical strength, especially for the oxide-based solid electrolyte. Compared with ISEs, SPEs, composed of uniform mixtures of polymer host and Li salt, have attracted much attention for their lightweight, high flexibility, and ease of processing. The main issues that hinder the application of SPEs are low room-temperature conductivity, and weak mechanical strength to suppress Li dendrite. To circumvent these problems, CPEs, combining SPEs with nanoparticle fillers, plasticizers, or 3D robust host, are considered more suitable as solid electrolyte [23,24,25,26,27]. CPE films with high room-temperature Li-ion conductivity and strong mechanical strength is the key for high performance SSBs. To achieve this goal, we studied two strategies to address the ionic conductivity and mechanical strength issues. (1) Succinonitrile (SN) plastic crystal is a non-ionic plastic material, with molecules ordered into a crystalline lattice, meanwhile exhibiting a fluctuational degree of freedom. In recent years, SN have been widely introduced into CPEs system as versatile additive to enhance the room-temperature Li-ion conductivity [28,29,30,31,32,33]. (2) In another side, to enhance the mechanical strength, many kinds of 3D frameworks have been studied as supporting matrix, including organic and inorganic networks [24,25,26]. Polyimide (PI) is an insulating material that has excellent thermal (>300 °C) and chemical stabilities. There are already many studies using PI as functional separator, as well as supporting matrix for SPEs [34,35,36,37,38,39]. Besides, PI owns a novel property of fireproof, which is very important for the safety of batteries.

In this work, we proposed a type of CPEs with high room-temperature Li-ion conductivity and good mechanical flexibility via UV polymerization method. In this CPE, SN is added as plasticizer and PI nano-fiber film is used as skeleton. The CPE film shows a very good flexibility, and a high room-temperature ionic conductivity of 2.64 × 10^−4^ S cm^−1^. A ASSLB cell assembled with LiCoO_2_ (LCO) cathode shows an initial discharge capacity of 131.1 mAh g^−1^, and 83.3% retention after 120 cycles. The CPE film also shows a novel fireproof property that enables promising safe ASSLBs.

## 2. Materials and Methods

### 2.1. Preparation of Polyimide (PI) Fiber

The PI fiber film was prepared via electrospinning deposition followed by an imidization process. In details, 4,4′-oxybisbenzenamine (ODA, ≥98%, Aladdin) was added to dimethylacetamide (DMAC, 99.9%, Sinopharm Chemical Reagent Co., Ltd., Shanghai, China) under magnetic stirring. Then, 1,2,4,5-Benzenetetracarboxylic anhydride (PMDA, ≥99%, Aladdin, Shanghai, China) was added after ODA completely dissolved, where the weight ratio of ODA to PMDA was 1:1, to get polyamide acid (PAA) solution after 12 h stirring. The concentration of PAA solution is 15 wt.%.

For electrospinning, the voltage was 20 kV, the distance between needle and collecting plate (roller collector 42BYGH47-401A, Alibaba Group, Hangzhou, China) was 18 cm and the propulsion speed was 1 ml h^−1^. Then PAA nanofiber membranes were dehydrated and cyclized at 100 °C for 2 h and 200 °C for 2 h and 300 °C for 2 h, respectively, in a muffle furnace to get the final product of PI nanofiber film.

### 2.2. Preparation of PI–CPE

LiTFSI (99%, Aladdin) and SN (99%, Macklin, Shanghai, China) were dissolved in PEGDA (~700, Macklin) in Ar filled glovebox with a mass ratio of 4:4:2 followed by magnetical stirring for 4 h, then 1 wt.% of photoinitiator CIBA (IRGACURE 819) (with respect to PEGDA) were added to obtain homogeneous precursor solution. To stabilize the interface between electrolyte and Li metal, 1 wt.% of LiNO_3_ (99.99%, Aladdin, with respect to total weight) was added to the precursor solution. The as-prepared precursor solution was dipped onto PI nanofiber film and allowed to stand for 5 min to let solution filtrated into the PI film. The obtained composite membrane was sandwiched between two glass slides and a UV-cured with UVLED machine (UVB 365 nm, XM210, Aventk, Shanghai, China). The prepared CPE film is named PI–CPE.

### 2.3. Characterization of Samples

The morphology of samples was characterized by a scanning electron microscopy (SEM, Phenom Pro X, Phenom Scientific, Shanghai, China). Fourier transform infrared spectrometer (ATR-FT-IR, IN10+IZ10, Nicolet, Madison, WI, USA) over the range from 400 to 4000 cm^−1^. Mechanical strength of PI-CPE was evaluated by a dynamic mechanical thermal analyzer (DMA, Q800, TA instruments, New Castle, DE, USA). The phase transition of materials during heating were investigated by differential scanning calorimeter (DSC, DSC822E, METTLER TOLEDO, Shanghai, China) performed from −100 °C to 150 °C.

### 2.4. Electrochemical Characterization

The PI–CPE film was evaluated as a solid electrolyte in stainless steel (SS)||SS, SS||Li, Li||Li, and Li||LiCoO_2_ (LCO) cell configurations. The ionic conductivity of PI-CPE was measured by Bio-Logic SP-500 with impedance spectra method of SS||SS cell over a frequency range between 7 MHz and 1 Hz at a temperature range from room temperature (RT) to 80 °C. The electrochemical window of the PI–CPE was tested by electrochemical floating analysis and linear sweep voltammetry (LSV) at a sweep rate of 1 mV s^−1^ from 0 to 6 V with carbon coated Al foil as the working electrode and Li metal as the counter electrode (C–Al||Li cell). The lithium ion transference number ($t_{{\mathrm{Li}}^{+}}$) of PI–CPE was evaluated by combining alternating current impedance and direct-current (DC) polarization with a DC voltage of 10mV using a Li||Li cell. The stability of PI–CPE with Li metal was carried out by Li plating/stripping cycles at a current density of 0.1 mA cm^−2^ with Li||Li symmetric cell on a LAND CT2001A testing system.

### 2.5. Evaluation of Solid-State Batteries

Cathode slurry was prepared by grinding LCO powder, carbon black, and polyvinylidene difluoride powder (PVDF) with mass fractions of 80%, 10%, and 10%, respectively, followed by the addition of methylpyrrolidone (NMP) solvent. The cathode slurry was then casted onto aluminum film, and dried in a vacuum oven at 80 °C for 12 h. Precursor electrolyte solution was dipped onto cathode film and kept for 2 h; then, the well-infiltrated cathode and PI film infiltrated with precursor solution were stacked together and in situ photo-polymerization. Coin cells were assembled with Li foil as anode and charging/discharging tests of batteries were performed on a LAND CT2001A testing system at room temperature.

## 3. Results

### 3.1. Structural Characterization of PI–CPE

**Figure 1:**
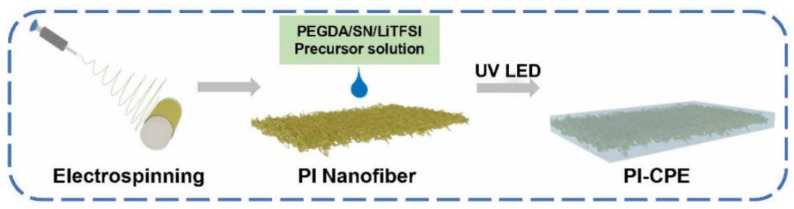
Schematic of the preparation process.

**Figure 2:**
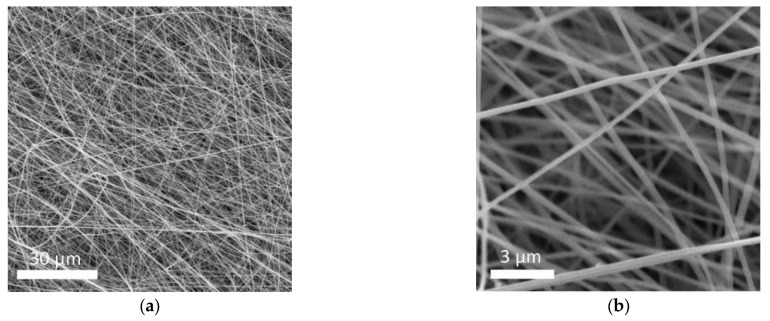
(**a**,**b**) are SEM images of PI nanofiber at different magnifications.

**Figure 3:**
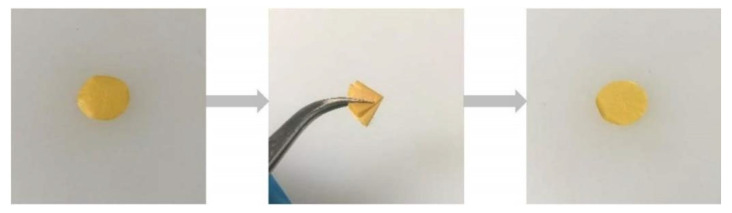
Twisting test of PI–CPE film.

The schematic in Figure 1 shows the preparation progress of PI–CPE. PI nanofiber obtained via electrospinning is firstly filled with precursor solution. Notably, there is no additional solvent existing in the precursor, and it is a solvent-free process. Under UV irradiation, PEDGA will be polymerized, and the precursor solution changes from liquid to solid. Figure S1 shows the photo image of the as-prepared PI film. The morphology of PI nanofiber is characterized by SEM, shown in Figure 2. The PI nanofibers all have a smooth surface, and much space formed the inner of the PI film caused by the stacking of PI fibers. So, PI-nanofiber film can serve as 3D framework to support the solid electrolyte. Figure S2 shows the photo image of PI–CPE film. The PI-CPE film has a very flexible nature. When put on a glass tube, PI–CPE can naturally droop due to the gravity. To further verify the flexibility of PI–CPE, a simple twisting test is performed (Figure 3). When PI––CPE recovers from twisting, it can regain its original morphology with a smooth surface, and no scratch appears on the surface.

**Figure 4:**
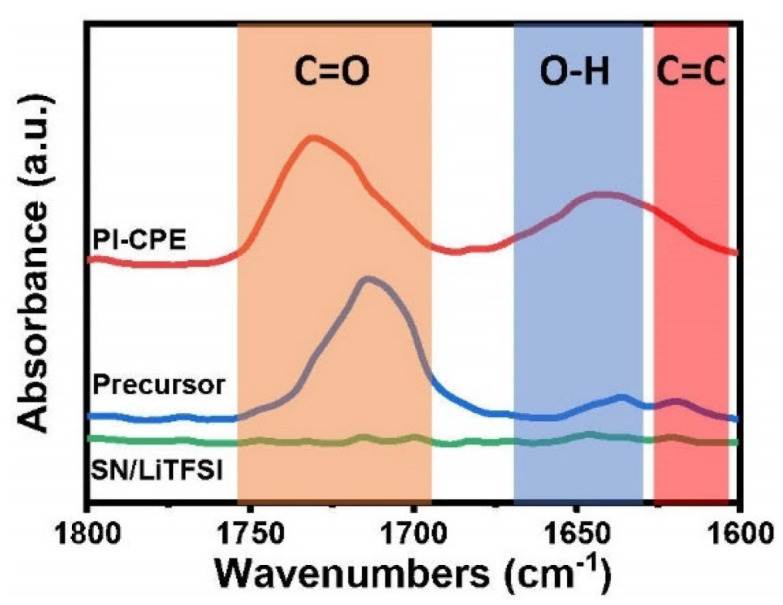
Fourier transform infrared (FTIR) spectra of PI–CPE.

The solidification of precursor solution under UV irradiation is contributed by the crosslinking of PEGDA molecule. To verify the reaction, FTIR of PI–CPE, precursor solution, and pure SN/LiTFSI without PEGDA are performed, and results are shown in Figure 4. Without PEGDA, spectra of SN/LiTFSI shows a flat line, and no peak can be seen at the wavenumber range from 1600 to 1800 cm^−1^. Compared with precursor solution, after UV polymerization, the characteristic peak of C=C at 1617 cm^−1^ of PI–CPE disappears, and the adsorption peak of C=O stretching at 1713 cm^−1^ blue shifts to 1731 cm^−1^, which confirms the breaking of C=C bonds and involvement of PEG back-bones. So, the PI–CPE has been well UV cured for a free-standing solid electrolyte. The peak is present at 1640 cm^−1^ with respect to the OH bending mode of water due to the absorption of moisture when exposed to air. 

**Figure 5:**
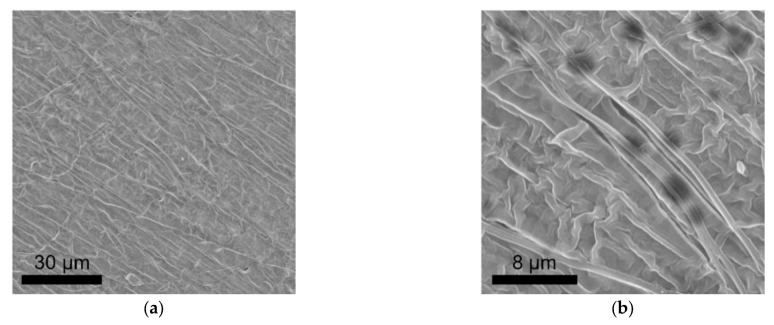
(**a**) and (**b**) are top-surface SEM images of PI–CPE at different magnifications.

**Figure 6:**
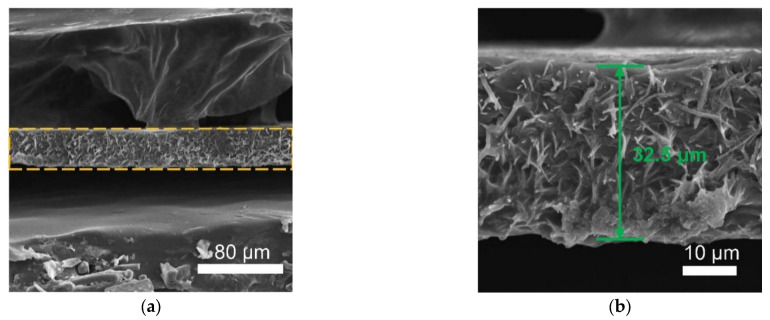
(**a**) and (**b**) are cross-sectional SEM images of PI–CPE at different magnifications.

After polymerization, the morphology of as the prepared PI–CPE is characterized with SEM. Figure 5a,b is the top-surface SEM images of PI–CPE, showing a dense structure and no PI fiber or holes can be found at the surface. To make sure the dense structure inside the PI–CPE, cross-section SEM images were also collected, as shown in Figure 6a,b. The inner of PI–CPE is fully filled with CPE with no pore structure. So, the dense structure of PI–CPE is further confirmed. The thickness of PI–CPE is about 32.5 μm. Figure S3 demonstrates the stress-strain curves of PI–CPE film. The tensile strength of PI–CPE can reach as high as 5.2 MPa with an elongation-at-break of 26%. A good mechanical strength can enable PI–CPE, suppressing the Li dendrite growth.

### 3.2. Electrochemical Performance

**Figure 7:**
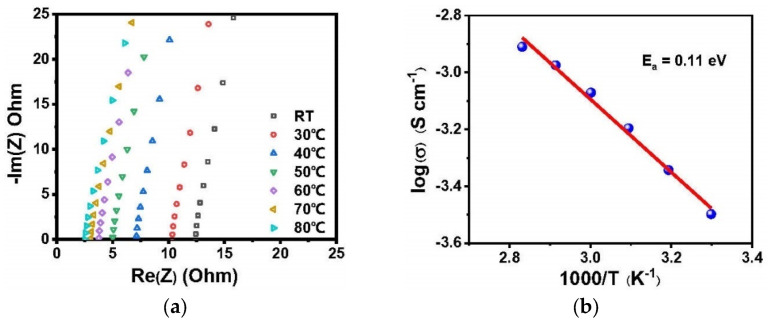
(**a**) EIS plots of PI–CPE at different temperatures; (**b**) Arrhenius plots of PI–CPE.

**Figure 8:**
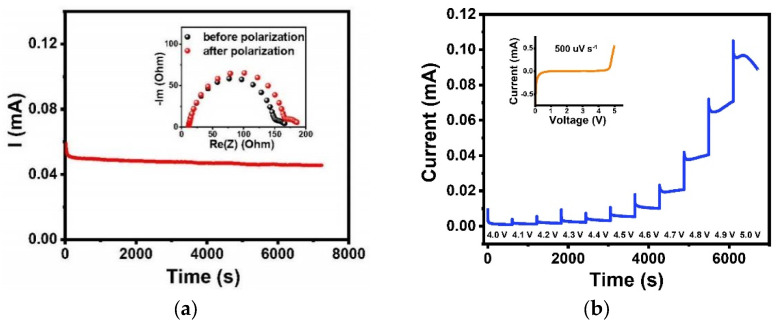
(**a**) Current-time curve and EIS plots before and after polarization for PI-CPE with Li||Li cell. (**b**) Electrochemical floating analysis with C–Al|PI–CPE|Li cell; inset is the LSV curve at a scan rate of 500 uV s^−1^.

Figure S4 shows the DSC curve of PI–CPE, and the low glass transition temperature of about −38.5 °C can benefit the room-temperature ionic conductivity. The ionic conductivity of PI–CPE is measured by SS||SS cell at a temperature range from RT to 80 °C, and EIS plots are shown in Figure 7a. The Li-ion conductivity of PI–CPE is as high as 2.64×10^−4^ S cm^−^^1^ at room temperature (25 °C). This high ionic conductivity combine with the relatively small thickness (32.5 μm) make PI–CPE qualified for practical application. The ionic conductivities of PI–CPE are 3.18 × 10^−4^ S cm^−^^1^, 4.54 × 10^−4^ S cm^−^^1^, 6.37 × 10^−4^ S cm^−^^1^, 8.49 × 10^−4^ S cm^−^^1^, 1.06 × 10^−3^ S cm^−^^1^, and 1.23 × 10^−3^ S cm^−^^1^ at 30 °C, 40 °C, 50 °C, 60 °C, 70 °C, and 80 °C, respectively. Figure 7b shows the Arrhenius plot, and the calculated activation energy E_a_ of PI–CPE by Arrhenius equation is 0.11 eV. For a solid electrolyte film, not only ionic conductivity but also Li-ion transference number ($t_{{\mathrm{Li}}^{+}}$ ) is a key factor that significant influent the cycle performance of SSBs. Too much anion transfer will cause polarization. DC polarization and alternating current impedance technology are employed together to measure $t_{{\mathrm{Li}}^{+}}$ of PI–CPE (Figure 8a). The $t_{{\mathrm{Li}}^{+}}$ of PI–CPE calculated by Bruce–Vincent–Evans equation is 0.76. It is much higher than the PEO-based polymer electrolyte, which is commonly around 0.2. Electrochemical window is another important parameter for SSEs. A wide electrochemical window can make SSEs compatible with high-voltage cathode, thus giving a higher energy density. Figure 8b shows the electrochemical floating analysis and LSV curve (inset) of PI–CPE, carbon-coated Al foil can enlarge the contact area between current collector and PI–CPE. Electrochemical floating analysis provides a stringent test of the oxidative stability of PI–CPE, and the electrochemical stable window is around 4.6 V, which is consistent with LSV test (Figure 8b).

**Figure 9:**
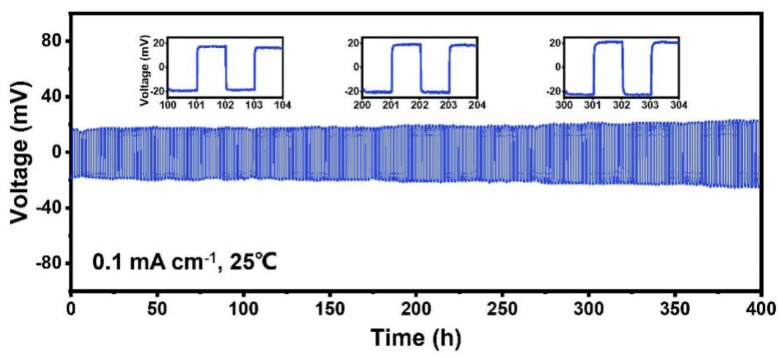
Cycling performance of Li|PI–CPE|Li symmetric cell.

**Figure 10:**
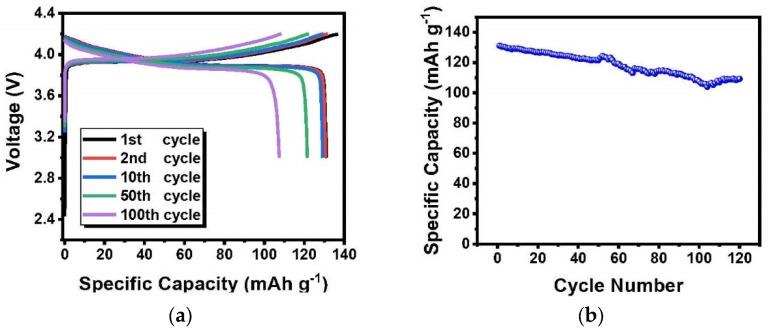
Voltage profile (**a**) and cycling performance (**b**) of PI–CPE at 0.2 C; cycling test was performed at room temperature.

The Li||Li symmetric cell is used to test whether this PI–CPE is compatible with the Li metal anode. Cycling test was carried out under a density of 0.1 mA cm^2^ and 1 h for each cycle at room temperature in Figure 9. The symmetric cell shows stable Li plating and stripping process. After cycling for 400 h, the overpotential only slightly increases from 18 mV to 24 mV. The good compatibility of PI–CPE with Li anode make it qualified for the SSBs. LCO|PI–CPE|Li coin cells are made to test the performance of PI–CPE film as SSE. LCO cathode is filled with the precursor solution same as PI–CPE and cured under a UV lamp. The LCO|PI–CPE|Li coin cell was cycled at room temperature. Figure 10a shows the voltage profile with a rate of 0.2 C. The initial charge and discharge specific capacity are 136.8 mAh g^−1^ and 131.1 mAh g^−1^, and the first-cycle coulombic efficiency is 95.8%. After 100 cycles, the over potential at the 100th cycle is no big difference with the 1st cycle, which shows good cycling stability. The LCO|PI–CPE|Li cell presents excellent cycling performance with 83.3% discharge capacity retention after 120 cycles at 0.2 C under room temperature. The high-voltage-compatible is also demonstrated in this test because of the use of LCO high voltage cathode.

### 3.3. Thermal and Fireproof Property

**Figure 11:**
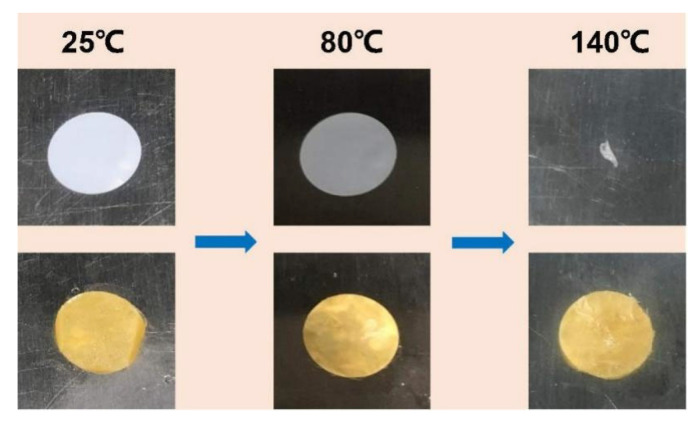
Comparison of thermal stability between commercial PP separator and PI–CPE.

**Figure 12:**
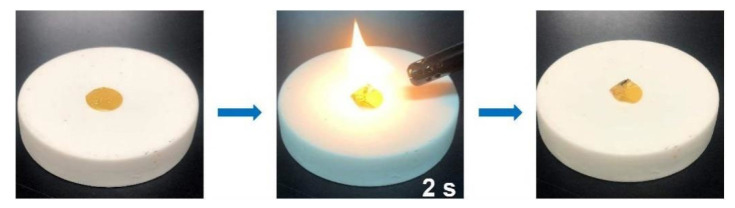
Flame test of PI–CPE.

Thermal stability is a very important parameter, which will reduce the risk of thermal runaway caused by short circuit. Commercial PP separator is used as comparison. PP and PI–CPE were heated from room temperature to 80 °C and 140 °C (Figure 11). From the pictures we can see that, when heated to 140 °C, PP shrinks to a point, and PI–CPE can still maintain its initial structure. The excellent stability of PI–CPE is mainly because the supporting matrix of PI fiber film. To test the fireproof property of PI–CPE, a flame test was also adopted. As shown in Figure 12, when exposed to the flame for 2 s, PI–CPE did not catch fire, showing a flame retardant property. Firstly, PI is highly thermally stable. Besides, SN will release CO, CO_2_, NO gas, etc., under high temperature, which can serve as fire extinguishing agent. So, the as-prepared PI–CPE is fireproof that is promising for high-safe SSBs.

## 4. Conclusions

We have proposed a flexible and fireproof polymer-polymer CPE thin film. Using SN as a plasticizer, the as-prepared PI–CPE shows a high room-temperature ionic conductivity of 2.64 × 10^−4^ S cm^−^^1^, which enables it suitable for SSBs running at ambient temperature. Due to the supporting of electrospun PI fiber, the PI–CPE has a high mechanical strength, giving an excellent ability suppressing Li dendrite growth. Moreover, the PI–CPE shows good thermal stability and is fireproof for SSBs with high safety. The Li|PI–CPE|Li symmetric cell can stable cycles more than 400 h, and the LCO|PI–CPE|Li full cell give an initial discharge specific capacity of 131.1 mAh g^−1^ with 83.3% discharge capacity retention after 120 cycles at 0.2 C under room temperature. This CPE is very promising for practical application of SSBs.

## Data Availability

Not applicable.

## Supplementary Materials

The following are available online at https://www.mdpi.com/article/10.3390/polym13213622/s1, Figure S1: Photograph of PI film, Figure S2: Photograph of PI–CPE film, Figure S3: Strain–stress curves of PI–CPE film, Figure S4: DSC curve of PI–CPE.
