# Supplementary material for: A Flexible, Fireproof, Composite Polymer Electrolyte Reinforced by Electrospun Polyimide for Room-Temperature Solid-State Batteries
Source: Polymers (Basel). 2021 Oct 20;13(21):3622. doi: 10.3390/polym13213622 (PMC8588480; doi:10.3390/polym13213622)
Supplement: Supplementary file 1 [file polymers-13-03622-s001.zip › polymers-1409391-supplementary.pdf]

An all-solid-state lithium-metal battery based on electrodes-compatible plastic crystal electrolyte working at ambient temperature

Bin Zhao [1,‡], *Boheng Yuan* [1,‡], *Zhi Cong* [1], *Zhi Cheng* [1], *Qi Wang* [1], *Yafei Lu* [1] *and Xiaogang Han* [1,2,*]

[1] State Key Laboratory of Electrical Insulation and Power Equipment, School of Electrical Engineering, Xi'an Jiaotong University, Xi'an, Shaanxi 710049, China

[2] Key Laboratory of Smart Grid of Shanxi Province, Xi'an, Shaanxi 710049, China

‡ Bin Zhao and Boheng Yuan contributed equally to this work

* Correspondence: xiaogang.han@xjtu.edu.cn

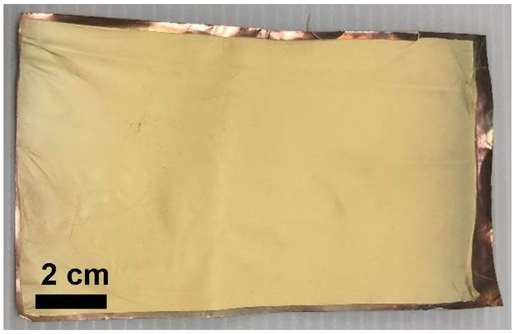

Figure S1. Photograph of PI film.

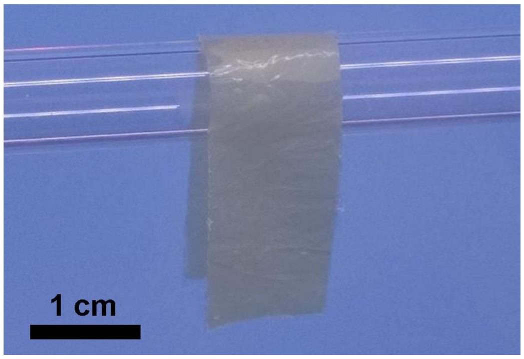

Figure S2. Photograph of PI-CPE film.

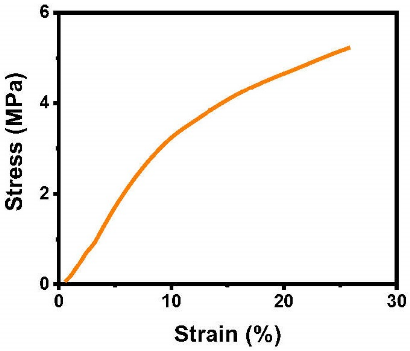

Figure S3. Strain–stress curves of PI-CPE film.

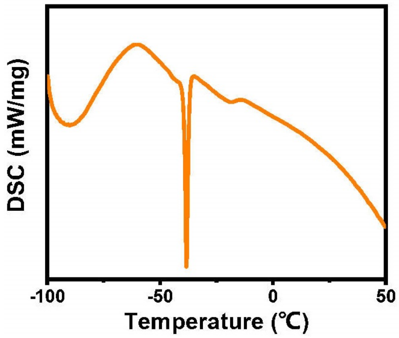

Figure S4. DSC curve of PI-CPE.
